# Crystal structures of *Z*–Gly–Aib–O^−^·0.5Ca^2+^·H_2_O and *Z*–Gly–Aib–OH

**DOI:** 10.1107/S2056989018010745

**Published:** 2018-07-27

**Authors:** Renate Gessmann, Hans Brückner, Kyriacos Petratos

**Affiliations:** a IMBB-FORTH, 70013 Heraklion, Greece; b Department of Food Sciences, Interdisciplinary Research Center, Justus-Liebig-University of Giessen, 35392 Giessen, Germany

**Keywords:** α-amino­isobutyric acid, dipeptide, peptide–calcium complex, hydrogen bonding, crystal structure

## Abstract

Both deprotonated and neutral achiral title dipeptides assume similar structures of two conformations, which are related by a unit-cell inversion centre. Two mol­ecules of both conformations of the metal-free neutral dipeptide are linked by two hydrogen bonds, while two mol­ecules of both conformations of the ionized form coordinate a calcium ion in calcium(II) bis­[2-(2-{[(benz­yl­oxy)carbon­yl]amino}­acetamido)-2-methyl­propano­ate] monohydrate, 0.5Ca^2+^·C_14_H_17_N_2_O_5_^−^·0.5H_2_O, which lies on an inversion centre and forms a distorted octa­hedral complex with the metal ion. These Ca^II^ complexes are connected in the crystal *via* hydrogen bonds in the *b*- and *c*-axis directions, whereas in the *a*-axis direction, they stack *via* apolar contacts. In the metal-free crystal, namely 2-(2-{[(benz­yloxy)carbon­yl]amino}­acetamido)-2-methyl­propanoic acid, C_14_H_18_N_2_O_5_, mol­ecules are hydrogen bonded in the *a*- and *c*-axis directions, and stack in the *b*–axis direction *via* apolar contacts.

## Abstract 2

Two deprotonated mol­ecules of *Z*–Gly–Aib^−^ form a complex with one Ca^II^ ion, which assumes a distorted octa­hedral conformation, whereas the respective metal-free, neutral and symmetry-inverted dipeptides *Z*–Gly–Aib–OH are mutually hydrogen-bonded.

## Chemical context   

The presence of Gly and Aib (α-amino­isobutyric acid) combines a residue with the greatest conformational flexibility (Gly) with a severely restricted residue (Aib) because of the second methyl group attached to the Cα atom. The space available for Aib comprises the left-handed and right-handed helical region of the Ramachandran plot. Because of the absent side-chain atoms, Gly can adopt almost all conformations in contrast to all other residues. This makes Gly a conserved residue in peptides and proteins because a mutation of Gly could change the flexibility necessary for function or cause significant alteration of the secondary structure. Gly is incorporated in about half of all known peptaibol sequences (Stoppacher *et al.*, 2013 ▸) and frequently as a –Aib–Gly– dipeptide or as a –Aib–Gly–Aib– tripeptide unit. Peptides composed of Aib and Gly only show an enormous structural flexibility (Gessmann *et al.*, 1991 ▸; Gessmann, Brückner, Aivaliotis *et al.*, 2015 ▸; Gessmann, Brückner & Petratos, 2015 ▸) and therefore normally normally do not yield suitable sized crystals for structure analysis with X-rays.
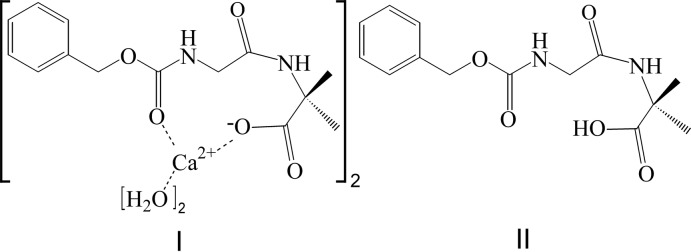


## Structural commentary   

In the crystal structure of **I** (*Z*–Gly–Aib-O^−^·0.5Ca^2+^·H_2_O) all expected non H-atoms in both dipeptides were readily visible in the first electron-density map as the highest peaks. In addition, a heavy atom was detected, which at a later stage was identified as calcium by energy-dispersive X-ray spectroscopy (EDS), together with a water oxygen atom.

**Figure 1:**
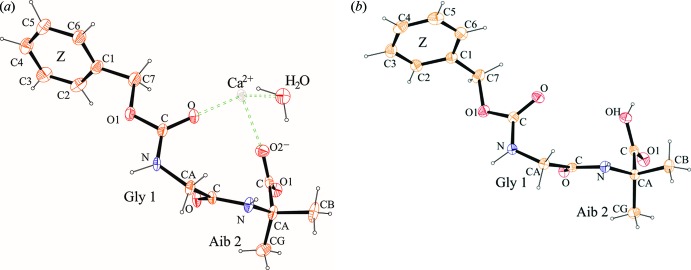
The mol­ecular structures of *Z*–Gly–Aib–OH showing the 50% probability displacement ellipsoids and simplified atom numbering (Farrugia, 2012 ▸). (*a*) The asymmetric unit of the complex with Ca^2+^ and H_2_O (I). One metal ion is coordinated by two symmetry-inverted peptides and water mol­ecules. (*b*) The structure of the neutral dipeptide *Z*-Gly-Aib-OH (II).

**Figure 2:**
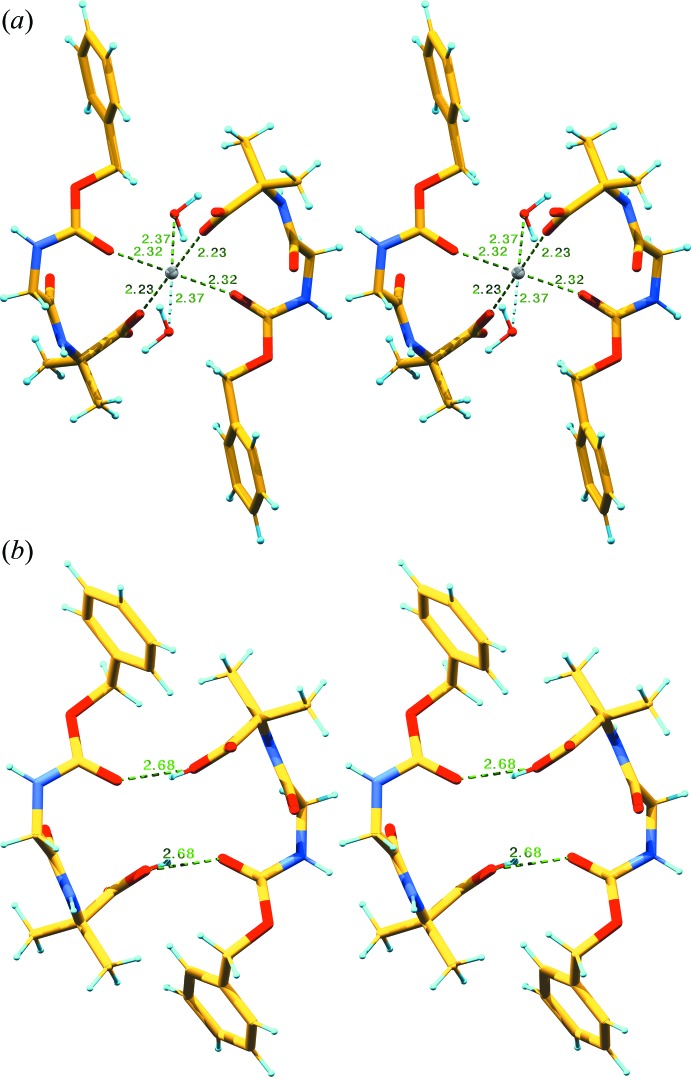
Wall-eyed stereo figure of two inversion-related mol­ecules of the metal-bound structure **I** (*a*) with the Ca^2+^ ion in grey and the free, neutral dipeptide **II** (*b*). Distances for the Ca^2+^ co-ordination (*a*) and hydrogen bonds (*b*) are shown in Å.

The backbone conformation of both peptides is very similar (Fig. 1 ▸). Gly is in the semi-extended conformation of both handednesses with torsion angles φ = ∓62.2 (2)°, ψ = ±153.37 (18)° in **I** and φ = ∓59.37 (14)°, ψ = ±153.66 (10)° in **II** (*Z*–Gly–Aib–OH). Aib adopts φ = ±54.8 (3) and ±55.86 (14)° in **I** and **II**, respectively, while the values of ψ with both O atoms are ∓154.7 (2) or ±29.7 (3)° in **I** and ∓145.5 (1) or ±41.0 (1)° in **II** and therefore lies in the helical region of the Ramachandran plot. The *Z*-protection groups (benzyl­oxycarbon­yl) adopt different conformations in **I** and **II** (Fig. 2 ▸). The r.m.s. deviation for the non-hydrogen atoms of Gly and Aib is 0.2 Å, whereby the most distant carbon atoms of the *Z* protection group of the two peptides are 4.75 Å apart in the superposition of the non-hydrogen atoms of the amino acid residues.

The similar backbone conformation is also visible in Fig. 2 ▸. Structure **I** crystallized with a water mol­ecule and a half calcium ion (lying on a special position) per peptide mol­ecule while **II**, which crystallized without any solvent mol­ecules, forms two direct hydrogen bonds between two inversion-related mol­ecules. In **I** the Ca^2+^ ion is coordinated by the carbonyl group of *Z* and the deprotonated carboxyl­ate group of Aib2. It is worth noting that in both crystal structures the same oxygen atoms participate in the hydrogen bonding and coordination inter­actions (Fig. 2 ▸). One dipeptide mol­ecule and its inverted mate provide four of the six ligand atoms for the calcium ion. The remaining two ligands are two water mol­ecules, which are also related *via* the inversion centres. The metal coordination parameters are listed in Table 1 ▸. As the calcium ion sits on the inversion centre, the values of the fifteen angles between the ligands are reduced to seven values, each one occurring twice and the 180° angle between the metal ion and inverted atoms occurs three times.

## Supra­molecular features   

**Figure 3:**
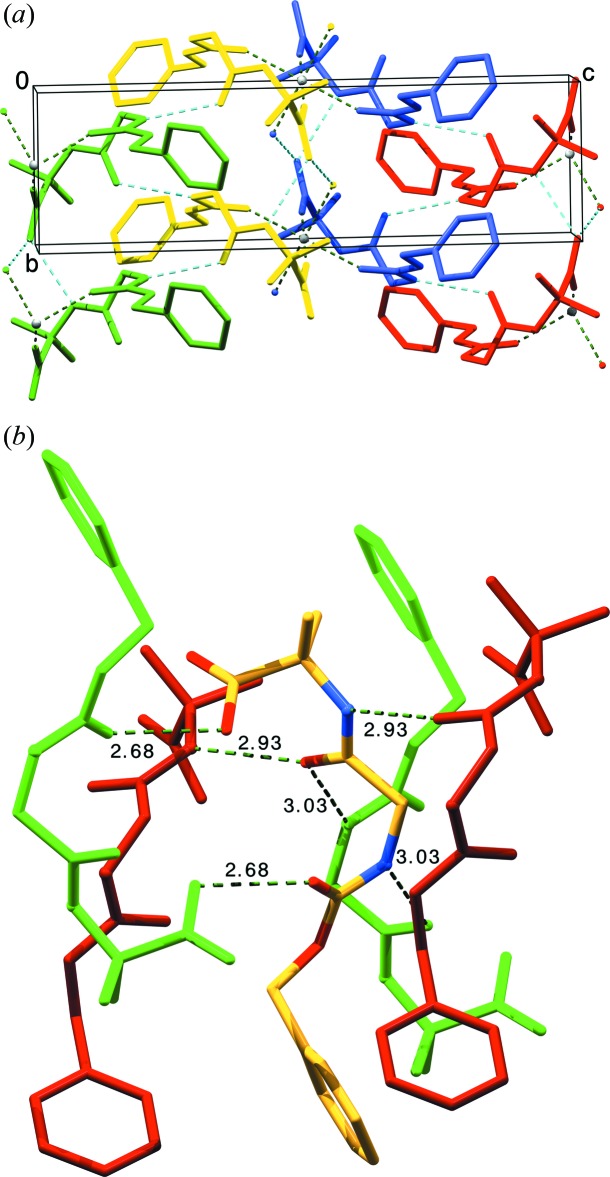
Mol­ecular packing of **I** and **II** showing the bonding to neighbouring mol­ecules. (*a*) In **I**, the Ca^2+^ ions are shown as a grey spheres at the inversion centres of the unit cell while the other spheres signify water mol­ecules. The content of two *y*-translated unit cells is shown. Hydrogen bonds are shown in cyan and the metal coordination bonds are shown in dark green. (*b*) The bonding of the central mol­ecule **II** (coloured atoms) to four neighbouring mol­ecules *via* hydrogen bonds.

**Figure 4:**
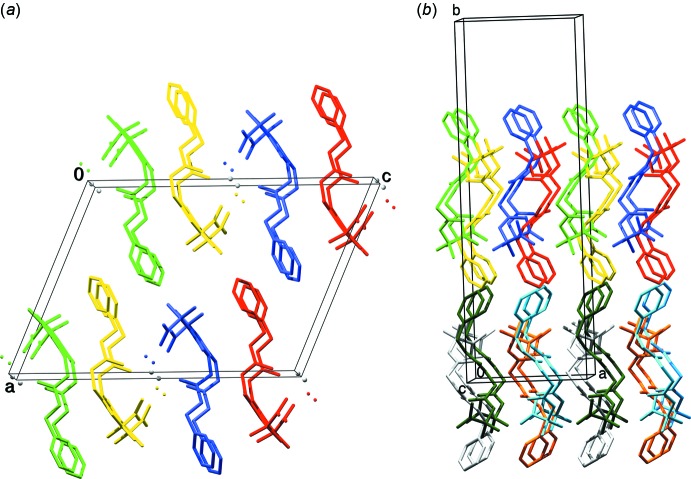
Mol­ecular packing of **I** and **II** showing the assembly in the crystal. (*a*) The content of two *x*- and two *y*-translated unit cells is shown. (*b*) The content of two *x*- and two *z*-translated unit cells is shown. The view is along the *b* axis in (*a*) and down the *c* axis in (*b*).

The crystal packing is quite different in **I** and **II**. In the crystal structure of **I**, there are four hydrogen bonds between symmetry-related mol­ecules (Table 2 ▸). The first hydrogen bond connects the NH group of Gly to the carbonyl group of a Gly residue, which belongs to a symmetry-related mol­ecule, *via* a screw axis. In Fig. 3 ▸ *a* the green and yellow mol­ecules are hydrogen bonded in a zigzag manner down the *b* axis and the blue and red symmetry-related ones in zigzag manner along the *b* axis. The second inter­molecular hydrogen bond is formed between the NH group of Aib and the carbonyl group of Aib of a *y*-translated (+1 or −1) mol­ecule, shown as pairs of the same color in Fig. 3 ▸ *a*. The same carbonyl group accepts the hydrogen bond from the water mol­ecule. As this water mol­ecule also coordinates the calcium ion, which is bonded to the translated and inverted mol­ecules, multiple bonded mol­ecule layers are formed in the *b*c plane. These single mol­ecule layers stack together *via* apolar contacts between the *Z* protection groups and the Aib side chains, along the *a*-axis direction (Fig. 4 ▸ *a*). The shortest distance between two symmetry-related rings is 3.54 Å and 3.91 Å between the Aib side chain and a symmetry-related ring. The staggering angles between the *Z* rings of successive sheets are 119.9° while the distance between the centre of the rings is 4.79 Å. Finally, in one layer the rings of the *Z* protection groups are staggered parallel with a distance of 5.57 Å, which is equal to the length of the *b* axis.

In the crystal structure of **II**, one mol­ecule (the left green mol­ecule in Fig. 3 ▸ *b*) is hydrogen bonded to four other mol­ecules. The carbonyl group of *Z* and the C-terminal OH group of Aib are hydrogen bonded to the same mol­ecule (Fig. 3 ▸ *b*) and to the left green mol­ecule. The NH group of Gly is hydrogen bonded to the carbonyl group of Gly1 of the right green mol­ecule in Fig. 3 ▸ *b* and Table 3 ▸. From the latter mol­ecule the NH-group of Gly1 is a hydrogen-bond donor to the carbonyl group of Gly1 of the central mol­ecule. The same carbonyl group of Gly1 is hydrogen bonded to the NH group of Aib2 of the left red mol­ecule, while the NH group of the central mol­ecule is hydrogen bonded to the carbonyl group of Aib2 of the right red mol­ecule in Fig. 3 ▸ *b*. From the same NH group of Aib2 there is a hydrogen-bonding distance of 3.31 Å to the carbonyl group of the same red mol­ecule. The N—H⋯O distance is 2.95 Å and the N—H⋯O angle is 107°, which are too long and too acute for hydrogen bonding; thus this carbonyl oxygen, which is the only potential hydrogen-bond former, remains non-bonded. Fig. 4 ▸ *b* shows all eight symmetry-related mol­ecules of the space group in different colors, zooming out from the central yellow mol­ecule in Fig. 3 ▸ *b*. Layers of hydrogen-bonded mol­ecules are formed in the *ac* plane, which stack together with the next layers along the *b* axis *via* apolar contacts. The rings of the *Z* groups inter­act between the layers through π–π stacking with an angle 120.1° and a distance between the centres of the rings of 5.73 Å. The shortest van der Waals distance between two layers is 3.86 Å, measured between two ring atoms of the *Z* protection groups. The staggering angles between the *Z* rings inside a sheet are 111.0° and the distance between the centres of the rings is 5.31 Å.

## Database survey   

The crystal structure of *t*-butyl­oxycarbon­yl–Gly–Aib–OH has been determined [CSD (Groom *et al.*, 2016 ▸) refcode CALFEA; Smith *et al.*, 1981 ▸]. The dipeptide assumes a different structure to the ones reported here. The N-terminal protection group points in opposite directions compared to the benzyl­oxycarbonyl (*Z*) of the present work. In addition, the crystal structure of an Aib containing peptide complexed with metal ions has also been determined, namely H–Aib–Gly–OH complexed with copper(II) (CSD refcode MUYNID; Tiliakos *et al.*, 2003 ▸). In this structure, one peptide takes part in the coordination of three copper ions and a metal ion is bonded to three peptides. This coordination is quite different from the one we have observed in the present work, where two peptide anions coordinate one calcium ion.

## Synthesis and crystallization   

The dipeptide *Z*–Gly–Aib–O^*t*^Bu (O^*t*^Bu, *tert*-but­oxy) was synthesized in DMF (di­methyl­formamide) from *Z*–Gly–OH (purchased from Bachem) and H–Aib–O^*t*^Bu using HOB^*t*^ and DCC (*N*,*N*′-di­cyclo­hexyl­carbodi­imide) as coupling reagents. *Z*–Gly–Aib–OH was obtained from *Z*–Gly–Aib–O^*t*^Bu with removal of the *tert*-butyl-protecting group by disolving in DCM (di­chloro­methane) and by adding TFA (tri­fluoro-acetic acid). The peptides were crystallized by slow evaporation from a methanol/water mixture (*v*:*v* = 50:50). Crystals of **I** and **II** were selected from different crystallization batches. In one crystallization batch, a small amount of calcium salt was present in the solvent, yielding the peptide–metal complex.

## Measurement and refinement   

Both crystals measured have a tiny third dimension. They were mounted on cryoloops without cryoprotectant and were kept in place with a minimal amount of vacuum grease and measured at 100 K. Photographs of the crystals are provided in Fig. S1 of the supporting information.

Diffraction data for the calcium-bound peptide (**I**) were collected on the microfocus beamline I24 (Evans *et al.*, 2011 ▸) of Diamond Light Source in Didcot, England, using a Pilatus3 6M detector (Dectris Ltd, Baden, Switzerland). A dataset of 1800 images covering 360° of rotation was collected in the resolution range 30.0–0.67 Å. 28517 reflections were recorded in total. Of these observed reflections, 4976 were unique. The data were integrated and scaled using the software package XDS (Kabsch, 2010 ▸). The initial space group *P*2_1_/*n* was changed to the conventional space group *P*2_1_/*c* with the *CCP4* programme suite (Winn *et al.*, 2011 ▸).

One single plate of the neutral peptide (**II**) was used for data collection at our in-house diffractometer and data were integrated and scaled with the Bruker software (Bruker, 2008 ▸). Crystal data, data collection and structure refinement details for both crystals are summarized in Table 4 ▸.

All non-hydrogen atoms and one water oxygen in **I** were detected in the direct methods solutions as highest peaks. The highest peak in **I** (583:220 to the second highest peak in relative units) in a special position was inter­preted from this height as a metal ion. The electron density of the metal ion pointed to more than double the number of electrons as oxygen and was assumed to be calcium. Additional supporting evidence came from the octa­hedral arrangement of six oxygen atoms around the metal. The central metal ion was unequiv­ocally identified as calcium *via* Energy-dispersive X-ray spectroscopy (EDS, Jeol Scanning Microscope 7000 F) by the occurrence of the characteristic peaks at 0.3 (*L*), 3.7 (*K*α) and 4.0 (*K*β) keV. The spectrum is shown in the supporting information section (Fig. S2).

## Supplementary Material

Crystal structure: contains datablock(s) I, II. DOI: 10.1107/S2056989018010745/ex2009sup1.cif

Structure factors: contains datablock(s) I. DOI: 10.1107/S2056989018010745/ex2009Isup2.hkl

Structure factors: contains datablock(s) II. DOI: 10.1107/S2056989018010745/ex2009IIsup3.hkl

Fig. S1 The crystals used for data collection. Fig. S2 Energy-dispersive X-ray emission spectrum.. DOI: 10.1107/S2056989018010745/ex2009sup4.pdf

CCDC references: 1833520, 1833521

Additional supporting information:  crystallographic information; 3D view; checkCIF report

## Figures and Tables

**Table 1:** Selected geometric parameters (Å, °) for **I** [Chem scheme1]

O—Ca_3	2.3200 (16)	Ca_3—O_4^i^	2.3702 (18)
O2_2—Ca_3	2.2343 (16)		
			
O^i^—Ca_3—O	180.0	O—Ca_3—O_4^i^	93.21 (6)
O—Ca_3—O2_2	94.24 (6)	O2_2—Ca_3—O_4	86.27 (7)
O^i^—Ca_3—O2_2	85.76 (6)	O2_2—Ca_3—O_4^i^	93.73 (7)
O—Ca_3—O_4	86.79 (6)		

Symmetry code: (i) 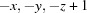.

**Table 2:** Hydrogen-bond geometry (Å, °) for **I** [Chem scheme1]

*D*—H⋯*A*	*D*—H	H⋯*A*	*D*⋯*A*	*D*—H⋯*A*
N_1—H_1⋯O_1^ii^	0.90 (3)	1.91 (3)	2.800 (2)	169 (3)
N_2—H_2⋯O1_2^iii^	0.73 (4)	2.18 (4)	2.864 (2)	156 (4)
O_4—H1_4⋯O1_2^iii^	1.00 (5)	1.75 (6)	2.741 (3)	172 (5)
O_4—H2_4⋯O^iv^	1.07 (6)	1.99 (6)	3.053 (2)	176 (6)

Symmetry codes: (ii) 
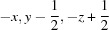
; (iii) 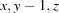; (iv) 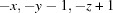.

**Table 3:** Hydrogen-bond geometry (Å, °) for **II** [Chem scheme1]

*D*—H⋯*A*	*D*—H	H⋯*A*	*D*⋯*A*	*D*—H⋯*A*
N_1—H_1⋯O_1^i^	0.92 (2)	2.12 (2)	3.0298 (14)	168.7 (16)
N_2—H_2⋯O_1^ii^	0.85 (2)	2.09 (2)	2.9304 (15)	167.3 (18)
O*H*_2—H*H*_2⋯O^iii^	0.88 (2)	1.82 (2)	2.6789 (14)	162 (2)

Symmetry codes: (i) 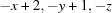; (ii) 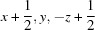; (iii) 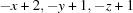.

**Table 4:** Experimental details

	**I**	**II**
Crystal data		
Chemical formula	0.5Ca^2+^·C_14_H_17_N_2_O_5_^−^·0.5H_2_O	C_14_H_18_N_2_O_5_
*M*_r_	331.35	294.30
Crystal system, space group	Monoclinic, *P*2_1_/*c*	Orthorhombic, *P* *b* *c* *a*
Temperature (K)	100	100
*a*, *b*, *c* (Å)	14.996 (3), 5.5740 (11), 20.607 (4)	9.5260 (19), 28.608 (6), 10.270 (2)
α, β, γ (°)	90, 112.55 (3), 90	90, 90, 90
*V* (Å^3^)	1590.8 (6)	2798.8 (10)
*Z*	4	8
Radiation type	Synchrotron, λ = 0.59038 Å	Cu *K*α
μ (mm^−1^)	0.14	0.90
Crystal size (mm)	0.18 × 0.06 × 0.03	0.2 × 0.1 × 0.05

Data collection		
Diffractometer	Pilatus3 6M detector on beamline I24 of Diamond Light Source	Bruker Venture D8
Absorption correction	–	Multi-scan (*SADABS*; Bruker, 2008 ▸)
*T*_min_, *T*_max_	–	0.90, 0.96
No. of measured, independent and observed [*I* > 2σ(*I*)] reflections	28071, 4716, 3941	44443, 2843, 2522
*R*_int_	0.128	0.067
(sin θ/λ)_max_ (Å^−1^)	0.750	0.634

Refinement		
*R*[*F* ^2^ > 2σ(*F* ^2^)], *wR*(*F* ^2^), *S*	0.078, 0.214, 1.06	0.036, 0.090, 1.09
No. of reflections	4716	2843
No. of parameters	279	262
H-atom treatment	H atoms treated by a mixture of independent and constrained refinement	All H-atom parameters refined
Δρ_max_, Δρ_min_ (e Å^−3^)	1.27, −0.56	0.27, −0.21

Computer programs: *PROTEUM2* and *SAINT* (Bruker, 2008 ▸), *XDS* (Kabsch, 2010 ▸), *SHELXS86* (Sheldrick, 2008 ▸), *SHELXL2014* (Sheldrick, 2015 ▸), *COOT* (Emsley *et al.*, 2010 ▸), *SwissPDBViewer* (Guex & Peitsch, 1997 ▸), *CHEMDRAW* (Mills, 2006 ▸), *ORTEPIII* (Burnett & Johnson, (1996 ▸), *ORTEP-3 for Windows* (Farrugia, 2012 ▸), *POVRAY* (Persistence of Vision, 2004 ▸), *pyMOL* (DeLano, 2002 ▸) and *publCIF* (Westrip, 2010 ▸).

## supplementary crystallographic information

### Calcium(II) bis[2-(2-{[(benzyloxy)carbonyl]amino}acetamido)-2-methylpropanoate] monohydrate (I) Crystal data

**Table:**

0.5Ca^2+^·C_14_H_17_N_2_O_5_^−^·0.5H_2_O	*F*(000) = 700
*M**_r_* = 331.35	*D*_x_ = 1.384 Mg m^−^^3^
Monoclinic, *P*2_1_/*c*	Synchrotron radiation, λ = 0.59038 Å
*a* = 14.996 (3) Å	Cell parameters from 4716 reflections
*b* = 5.5740 (11) Å	θ = 0.7–30°
*c* = 20.607 (4) Å	µ = 0.14 mm^−^^1^
β = 112.55 (3)°	*T* = 100 K
*V* = 1590.8 (6) Å^3^	Brick, colourless
*Z* = 4	0.18 × 0.06 × 0.03 mm

### Calcium(II) bis[2-(2-{[(benzyloxy)carbonyl]amino}acetamido)-2-methylpropanoate] monohydrate (I) Data collection

**Table:**

Pilatus3 6M detector on beamline I24 of Diamond Light Source diffractometer	*R*_int_ = 0.128
φ–scans	θ_max_ = 26.3°, θ_min_ = 1.2°
28071 measured reflections	*h* = −19→19
4716 independent reflections	*k* = −7→7
3941 reflections with *I* > 2σ(*I*)	*l* = −29→29

### Calcium(II) bis[2-(2-{[(benzyloxy)carbonyl]amino}acetamido)-2-methylpropanoate] monohydrate (I) Refinement

**Table:**

Refinement on *F*^2^	0 restraints
Least-squares matrix: full	Hydrogen site location: difference Fourier map
*R*[*F*^2^ > 2σ(*F*^2^)] = 0.078	H atoms treated by a mixture of independent and constrained refinement
*wR*(*F*^2^) = 0.214	*w* = 1/[σ^2^(*F*_o_^2^) + (0.1201*P*)^2^ + 1.1073*P*] where *P* = (*F*_o_^2^ + 2*F*_c_^2^)/3
*S* = 1.06	(Δ/σ)_max_ < 0.001
4716 reflections	Δρ_max_ = 1.27 e Å^−^^3^
279 parameters	Δρ_min_ = −0.56 e Å^−^^3^

### Calcium(II) bis[2-(2-{[(benzyloxy)carbonyl]amino}acetamido)-2-methylpropanoate] monohydrate (I) Special details

**Table:**

Geometry. All esds (except the esd in the dihedral angle between two l.s. planes) are estimated using the full covariance matrix. The cell esds are taken into account individually in the estimation of esds in distances, angles and torsion angles; correlations between esds in cell parameters are only used when they are defined by crystal symmetry. An approximate (isotropic) treatment of cell esds is used for estimating esds involving l.s. planes.

### Calcium(II) bis[2-(2-{[(benzyloxy)carbonyl]amino}acetamido)-2-methylpropanoate] monohydrate (I) Fractional atomic coordinates and isotropic or equivalent isotropic displacement parameters (Å^2^)

**Table:**

	*x*	*y*	*z*	*U*_iso_*/*U*_eq_	
C1	−0.31673 (15)	−0.0754 (5)	0.26150 (12)	0.0259 (5)	
C2	−0.33650 (17)	0.1220 (4)	0.21663 (15)	0.0312 (5)	
H2	−0.285 (2)	0.245 (6)	0.2275 (17)	0.030 (8)*	
C3	−0.42513 (18)	0.1428 (4)	0.16126 (15)	0.0310 (5)	
H3	−0.435 (2)	0.268 (6)	0.1291 (17)	0.026 (7)*	
C4	−0.49525 (17)	−0.0319 (5)	0.15046 (14)	0.0291 (5)	
H4	−0.558 (2)	−0.007 (6)	0.1123 (19)	0.032 (8)*	
C5	−0.47630 (16)	−0.2259 (5)	0.19555 (14)	0.0294 (5)	
H5	−0.527 (2)	−0.367 (6)	0.1923 (18)	0.033 (8)*	
C6	−0.38720 (16)	−0.2489 (5)	0.25022 (13)	0.0276 (5)	
H6	−0.373 (3)	−0.377 (8)	0.283 (2)	0.049 (11)*	
C7	−0.21987 (17)	−0.1049 (5)	0.31939 (13)	0.0315 (5)	
H71	−0.212 (2)	−0.252 (6)	0.3567 (19)	0.036 (8)*	
H72	−0.188 (3)	0.053 (7)	0.343 (2)	0.039 (9)*	
O1	−0.15523 (11)	−0.1822 (3)	0.28613 (8)	0.0257 (4)	
C	−0.06325 (14)	−0.2165 (4)	0.32957 (10)	0.0191 (4)	
O	−0.03479 (11)	−0.1969 (3)	0.39390 (8)	0.0225 (3)	
N_1	−0.00723 (12)	−0.2754 (3)	0.29610 (9)	0.0172 (3)	
H_1	−0.033 (2)	−0.300 (6)	0.2494 (18)	0.027 (7)*	
CA_1	0.09504 (14)	−0.3025 (4)	0.33509 (11)	0.0182 (4)	
HA1_1	0.1043 (18)	−0.429 (5)	0.3668 (15)	0.014 (6)*	
HA2_1	0.125 (2)	−0.342 (6)	0.3040 (17)	0.025 (7)*	
C_1	0.14182 (13)	−0.0694 (3)	0.37032 (10)	0.0157 (4)	
O_1	0.10901 (11)	0.1280 (3)	0.34679 (8)	0.0207 (3)	
N_2	0.22149 (12)	−0.0984 (3)	0.42780 (9)	0.0179 (3)	
H_2	0.229 (3)	−0.223 (8)	0.440 (2)	0.043 (10)*	
CA_2	0.27454 (14)	0.1042 (4)	0.47072 (11)	0.0197 (4)	
CB_2	0.34920 (18)	−0.0022 (4)	0.53830 (14)	0.0303 (6)	
HB1_2	0.388 (2)	−0.101 (5)	0.5219 (16)	0.021 (7)*	
HB2_2	0.316 (2)	−0.089 (5)	0.5596 (15)	0.018 (6)*	
HB3_2	0.385 (3)	0.117 (8)	0.571 (2)	0.055 (11)*	
CG_2	0.32397 (17)	0.2482 (4)	0.43140 (14)	0.0268 (5)	
HG1_2	0.366 (2)	0.152 (6)	0.4210 (17)	0.027 (7)*	
HG2_2	0.283 (2)	0.306 (6)	0.3910 (18)	0.030 (8)*	
HG3_2	0.361 (3)	0.369 (7)	0.463 (2)	0.042 (9)*	
C_2	0.20651 (14)	0.2604 (4)	0.49373 (10)	0.0184 (4)	
O1_2	0.22838 (12)	0.4764 (3)	0.50765 (9)	0.0242 (3)	
O2_2	0.13732 (12)	0.1601 (3)	0.50138 (9)	0.0259 (4)	
Ca_3	0.0000	0.0000	0.5000	0.01693 (17)	
O_4	0.09395 (13)	−0.3401 (3)	0.55420 (9)	0.0267 (4)	
H1_4	0.148 (4)	−0.398 (10)	0.541 (3)	0.079 (15)*	
H2_4	0.071 (4)	−0.503 (10)	0.570 (3)	0.095*	

### Calcium(II) bis[2-(2-{[(benzyloxy)carbonyl]amino}acetamido)-2-methylpropanoate] monohydrate (I) Atomic displacement parameters (Å^2^)

**Table:**

	*U*^11^	*U*^22^	*U*^33^	*U*^12^	*U*^13^	*U*^23^
C1	0.0171 (9)	0.0340 (12)	0.0251 (10)	0.0014 (8)	0.0064 (8)	−0.0075 (9)
C2	0.0234 (10)	0.0269 (11)	0.0442 (15)	−0.0030 (8)	0.0140 (10)	−0.0072 (10)
C3	0.0266 (11)	0.0263 (11)	0.0388 (13)	0.0048 (8)	0.0111 (10)	0.0031 (10)
C4	0.0221 (10)	0.0285 (11)	0.0311 (12)	0.0046 (8)	0.0041 (9)	−0.0030 (9)
C5	0.0203 (10)	0.0299 (11)	0.0340 (12)	−0.0023 (8)	0.0059 (9)	−0.0061 (9)
C6	0.0224 (10)	0.0313 (12)	0.0267 (11)	0.0013 (8)	0.0067 (9)	−0.0006 (9)
C7	0.0197 (10)	0.0482 (15)	0.0251 (11)	0.0010 (9)	0.0070 (9)	−0.0115 (10)
O1	0.0161 (7)	0.0405 (9)	0.0164 (7)	0.0025 (6)	0.0018 (6)	−0.0046 (6)
C	0.0173 (9)	0.0199 (9)	0.0154 (8)	−0.0030 (6)	0.0010 (7)	−0.0015 (7)
O	0.0214 (7)	0.0287 (8)	0.0137 (7)	−0.0026 (5)	0.0026 (5)	−0.0029 (6)
N_1	0.0151 (7)	0.0202 (8)	0.0099 (7)	−0.0005 (5)	−0.0021 (6)	−0.0024 (6)
CA_1	0.0155 (8)	0.0174 (9)	0.0160 (8)	0.0001 (6)	−0.0003 (7)	−0.0048 (7)
C_1	0.0171 (8)	0.0152 (8)	0.0112 (7)	−0.0010 (6)	0.0016 (6)	−0.0017 (6)
O_1	0.0245 (7)	0.0154 (7)	0.0137 (6)	0.0009 (5)	−0.0022 (5)	0.0020 (5)
N_2	0.0178 (7)	0.0106 (7)	0.0175 (8)	−0.0007 (5)	−0.0018 (6)	0.0010 (6)
CA_2	0.0169 (8)	0.0154 (8)	0.0188 (9)	−0.0015 (6)	−0.0019 (7)	0.0005 (7)
CB_2	0.0233 (11)	0.0206 (10)	0.0292 (12)	−0.0004 (7)	−0.0097 (9)	0.0016 (8)
CG_2	0.0231 (10)	0.0229 (10)	0.0349 (12)	−0.0037 (8)	0.0118 (9)	−0.0022 (9)
C_2	0.0198 (9)	0.0178 (8)	0.0116 (8)	−0.0025 (6)	−0.0006 (7)	0.0020 (6)
O1_2	0.0279 (8)	0.0148 (7)	0.0284 (8)	−0.0012 (5)	0.0090 (7)	−0.0002 (6)
O2_2	0.0265 (8)	0.0286 (8)	0.0240 (8)	−0.0100 (6)	0.0113 (6)	−0.0071 (6)
Ca_3	0.0189 (3)	0.0166 (3)	0.0123 (3)	−0.00324 (17)	0.0026 (2)	−0.00157 (17)
O_4	0.0304 (8)	0.0194 (7)	0.0290 (8)	0.0002 (6)	0.0101 (7)	0.0000 (6)

### Calcium(II) bis[2-(2-{[(benzyloxy)carbonyl]amino}acetamido)-2-methylpropanoate] monohydrate (I) Geometric parameters (Å, º)

**Table:**

C1—C6	1.385 (3)	CA_1—HA2_1	0.94 (3)
C1—C2	1.394 (4)	C_1—O_1	1.227 (2)
C1—C7	1.494 (3)	C_1—N_2	1.331 (2)
C2—C3	1.385 (4)	N_2—CA_2	1.467 (3)
C2—H2	0.99 (3)	N_2—H_2	0.73 (4)
C3—C4	1.387 (4)	CA_2—CG_2	1.520 (3)
C3—H3	0.94 (3)	CA_2—CB_2	1.533 (3)
C4—C5	1.382 (4)	CA_2—C_2	1.547 (3)
C4—H4	0.98 (3)	CB_2—HB1_2	0.95 (3)
C5—C6	1.384 (3)	CB_2—HB2_2	0.92 (3)
C5—H5	1.08 (3)	CB_2—HB3_2	0.96 (5)
C6—H6	0.95 (4)	CG_2—HG1_2	0.91 (3)
C7—O1	1.451 (3)	CG_2—HG2_2	0.89 (3)
C7—H71	1.10 (4)	CG_2—HG3_2	0.96 (4)
C7—H72	1.03 (4)	C_2—O2_2	1.241 (3)
O1—C	1.339 (2)	C_2—O1_2	1.252 (2)
C—O	1.232 (2)	O2_2—Ca_3	2.2343 (16)
C—N_1	1.317 (3)	Ca_3—O2_2^i^	2.2342 (16)
O—Ca_3	2.3200 (16)	Ca_3—O^i^	2.3200 (16)
N_1—CA_1	1.441 (2)	Ca_3—O_4^i^	2.3702 (18)
N_1—H_1	0.90 (3)	Ca_3—O_4	2.3702 (17)
CA_1—C_1	1.522 (3)	O_4—H1_4	1.00 (5)
CA_1—HA1_1	0.93 (3)	O_4—H2_4	1.07 (6)
			
C6—C1—C2	119.0 (2)	C_1—N_2—H_2	113 (3)
C6—C1—C7	120.2 (2)	CA_2—N_2—H_2	123 (3)
C2—C1—C7	120.7 (2)	N_2—CA_2—CG_2	110.34 (19)
C3—C2—C1	120.4 (2)	N_2—CA_2—CB_2	106.75 (16)
C3—C2—H2	122.9 (19)	CG_2—CA_2—CB_2	110.68 (19)
C1—C2—H2	116.7 (19)	N_2—CA_2—C_2	110.43 (16)
C2—C3—C4	120.1 (2)	CG_2—CA_2—C_2	112.33 (17)
C2—C3—H3	119.1 (19)	CB_2—CA_2—C_2	106.08 (19)
C4—C3—H3	121 (2)	CA_2—CB_2—HB1_2	103.5 (18)
C5—C4—C3	119.6 (2)	CA_2—CB_2—HB2_2	107.3 (18)
C5—C4—H4	122.3 (19)	HB1_2—CB_2—HB2_2	112 (3)
C3—C4—H4	118.0 (19)	CA_2—CB_2—HB3_2	113 (3)
C4—C5—C6	120.3 (2)	HB1_2—CB_2—HB3_2	113 (3)
C4—C5—H5	124.7 (18)	HB2_2—CB_2—HB3_2	107 (3)
C6—C5—H5	115.0 (18)	CA_2—CG_2—HG1_2	110 (2)
C1—C6—C5	120.5 (2)	CA_2—CG_2—HG2_2	113 (2)
C1—C6—H6	117 (2)	HG1_2—CG_2—HG2_2	107 (3)
C5—C6—H6	122 (2)	CA_2—CG_2—HG3_2	107 (2)
O1—C7—C1	106.06 (19)	HG1_2—CG_2—HG3_2	107 (3)
O1—C7—H71	101.6 (18)	HG2_2—CG_2—HG3_2	114 (3)
C1—C7—H71	116.7 (18)	O2_2—C_2—O1_2	124.2 (2)
O1—C7—H72	102 (2)	O2_2—C_2—CA_2	117.83 (18)
C1—C7—H72	115 (2)	O1_2—C_2—CA_2	117.76 (18)
H71—C7—H72	113 (3)	C_2—O2_2—Ca_3	171.82 (15)
C—O1—C7	115.48 (17)	O2_2^i^—Ca_3—O2_2	180.0
O—C—N_1	123.95 (19)	O2_2^i^—Ca_3—O^i^	94.24 (6)
O—C—O1	123.3 (2)	O2_2—Ca_3—O^i^	85.75 (6)
N_1—C—O1	112.70 (17)	O^i^—Ca_3—O	180.0
C—O—Ca_3	156.34 (15)	O—Ca_3—O2_2	94.24 (6)
C—N_1—CA_1	119.38 (17)	O^i^—Ca_3—O2_2	85.76 (6)
C—N_1—H_1	120 (2)	O—Ca_3—O_4	86.79 (6)
CA_1—N_1—H_1	121 (2)	O—Ca_3—O_4^i^	93.21 (6)
N_1—CA_1—C_1	111.96 (16)	O2_2—Ca_3—O_4	86.27 (7)
N_1—CA_1—HA1_1	108.0 (16)	O2_2—Ca_3—O_4^i^	93.73 (7)
C_1—CA_1—HA1_1	113.1 (17)	O2_2^i^—Ca_3—O_4	93.73 (7)
N_1—CA_1—HA2_1	109.4 (18)	O2_2—Ca_3—O_4	86.27 (7)
C_1—CA_1—HA2_1	105.6 (19)	O^i^—Ca_3—O_4	93.21 (6)
HA1_1—CA_1—HA2_1	109 (3)	O—Ca_3—O_4	86.79 (6)
O_1—C_1—N_2	123.21 (18)	O_4^i^—Ca_3—O_4	180.0
O_1—C_1—CA_1	122.38 (17)	Ca_3—O_4—H1_4	122 (3)
N_2—C_1—CA_1	114.41 (17)	Ca_3—O_4—H2_4	128 (3)
C_1—N_2—CA_2	122.52 (17)	H1_4—O_4—H2_4	101 (4)

Symmetry code: (i) −*x*, −*y*, −*z*+1.

### Calcium(II) bis[2-(2-{[(benzyloxy)carbonyl]amino}acetamido)-2-methylpropanoate] monohydrate (I) Hydrogen-bond geometry (Å, º)

**Table:**

*D*—H···*A*	*D*—H	H···*A*	*D*···*A*	*D*—H···*A*
N_1—H_1···O_1^ii^	0.90 (3)	1.91 (3)	2.800 (2)	169 (3)
N_2—H_2···O1_2^iii^	0.73 (4)	2.18 (4)	2.864 (2)	156 (4)
O_4—H1_4···O1_2^iii^	1.00 (5)	1.75 (6)	2.741 (3)	172 (5)
O_4—H2_4···O^iv^	1.07 (6)	1.99 (6)	3.053 (2)	176 (6)

Symmetry codes: (ii) −*x*, *y*−1/2, −*z*+1/2; (iii) *x*, *y*−1, *z*; (iv) −*x*, −*y*−1, −*z*+1.

### 2-(2-{[(Benzyloxy)carbonyl]amino}acetamido)-2-methylpropanoic acid (II) Crystal data

**Table:**

C_14_H_18_N_2_O_5_	*D*_x_ = 1.397 Mg m^−^^3^
*M**_r_* = 294.30	Cu *K*α radiation, λ = 1.54178 Å
Orthorhombic, *P**b**c**a*	Cell parameters from 312 reflections
*a* = 9.5260 (19) Å	θ = 3.1–44.6°
*b* = 28.608 (6) Å	µ = 0.90 mm^−^^1^
*c* = 10.270 (2) Å	*T* = 100 K
*V* = 2798.8 (10) Å^3^	Plate, colourless
*Z* = 8	0.2 × 0.1 × 0.05 mm
*F*(000) = 1248	

### 2-(2-{[(Benzyloxy)carbonyl]amino}acetamido)-2-methylpropanoic acid (II) Data collection

**Table:**

Bruker Venture D8 diffractometer	2522 reflections with *I* > 2σ(*I*)
profile data from φ or ω scans	*R*_int_ = 0.067
Absorption correction: multi-scan (SADABS; Bruker, 2008)	θ_max_ = 77.7°, θ_min_ = 3.1°
*T*_min_ = 0.90, *T*_max_ = 0.96	*h* = −11→12
44443 measured reflections	*k* = −36→36
2843 independent reflections	*l* = −12→13

### 2-(2-{[(Benzyloxy)carbonyl]amino}acetamido)-2-methylpropanoic acid (II) Refinement

**Table:**

Refinement on *F*^2^	0 restraints
Least-squares matrix: full	Hydrogen site location: difference Fourier map
*R*[*F*^2^ > 2σ(*F*^2^)] = 0.036	All H-atom parameters refined
*wR*(*F*^2^) = 0.090	*w* = 1/[σ^2^(*F*_o_^2^) + (0.0396*P*)^2^ + 1.1904*P*] where *P* = (*F*_o_^2^ + 2*F*_c_^2^)/3
*S* = 1.09	(Δ/σ)_max_ = 0.001
2843 reflections	Δρ_max_ = 0.27 e Å^−^^3^
262 parameters	Δρ_min_ = −0.21 e Å^−^^3^

### 2-(2-{[(Benzyloxy)carbonyl]amino}acetamido)-2-methylpropanoic acid (II) Special details

**Table:**

Geometry. All esds (except the esd in the dihedral angle between two l.s. planes) are estimated using the full covariance matrix. The cell esds are taken into account individually in the estimation of esds in distances, angles and torsion angles; correlations between esds in cell parameters are only used when they are defined by crystal symmetry. An approximate (isotropic) treatment of cell esds is used for estimating esds involving l.s. planes.

### 2-(2-{[(Benzyloxy)carbonyl]amino}acetamido)-2-methylpropanoic acid (II) Fractional atomic coordinates and isotropic or equivalent isotropic displacement parameters (Å^2^)

**Table:**

	*x*	*y*	*z*	*U*_iso_*/*U*_eq_	
C1	0.96843 (14)	0.33651 (5)	0.17611 (13)	0.0204 (3)	
C2	0.91669 (14)	0.31322 (5)	0.06711 (13)	0.0220 (3)	
H2	0.8387 (18)	0.3269 (6)	0.0193 (16)	0.027 (4)*	
C3	0.97610 (15)	0.27138 (5)	0.02714 (14)	0.0257 (3)	
H3	0.9404 (18)	0.2549 (6)	−0.0516 (17)	0.030 (4)*	
C4	1.08684 (15)	0.25222 (5)	0.09657 (16)	0.0287 (3)	
H4	1.130 (2)	0.2242 (7)	0.0670 (18)	0.042 (5)*	
C5	1.13629 (16)	0.27456 (5)	0.20693 (15)	0.0302 (3)	
H5	1.2082 (19)	0.2620 (6)	0.2553 (18)	0.031 (4)*	
C6	1.07709 (15)	0.31656 (5)	0.24705 (14)	0.0259 (3)	
H6	1.1110 (17)	0.3317 (6)	0.3233 (16)	0.024 (4)*	
C7	0.90948 (16)	0.38351 (5)	0.21154 (15)	0.0262 (3)	
H71	0.810 (2)	0.3851 (6)	0.1938 (16)	0.026 (4)*	
H72	0.9308 (18)	0.3923 (6)	0.3014 (18)	0.030 (4)*	
O1	0.96954 (10)	0.41880 (3)	0.12554 (9)	0.0222 (2)	
C	1.06743 (13)	0.44778 (4)	0.17393 (12)	0.0163 (3)	
O	1.10316 (10)	0.45014 (3)	0.28856 (9)	0.0215 (2)	
N_1	1.12335 (11)	0.47415 (4)	0.07933 (10)	0.0158 (2)	
H_1	1.0918 (19)	0.4703 (6)	−0.0046 (19)	0.033 (5)*	
CA_1	1.20786 (12)	0.51416 (4)	0.11432 (12)	0.0161 (2)	
H1_1	1.2928 (17)	0.5050 (5)	0.1588 (15)	0.017 (4)*	
H2_1	1.2366 (16)	0.5302 (6)	0.0336 (15)	0.020 (4)*	
C_1	1.12366 (12)	0.54897 (4)	0.19605 (11)	0.0138 (2)	
O_1	0.99489 (9)	0.55194 (3)	0.18472 (8)	0.0163 (2)	
N_2	1.19880 (11)	0.57585 (4)	0.27582 (10)	0.0147 (2)	
H_2	1.287 (2)	0.5720 (6)	0.2796 (16)	0.026 (4)*	
CA_2	1.13637 (12)	0.61511 (4)	0.34700 (12)	0.0153 (2)	
CB_2	1.24310 (14)	0.63395 (5)	0.44538 (13)	0.0209 (3)	
HB1_2	1.2728 (17)	0.6095 (6)	0.5053 (16)	0.026 (4)*	
HB2_2	1.200 (2)	0.6593 (7)	0.4966 (17)	0.036 (5)*	
HB3_2	1.3238 (19)	0.6467 (6)	0.4009 (16)	0.028 (4)*	
CG_2	1.09477 (15)	0.65365 (5)	0.25267 (14)	0.0220 (3)	
HG1_2	1.0301 (19)	0.6422 (6)	0.1863 (16)	0.029 (4)*	
HG2_2	1.0499 (19)	0.6787 (7)	0.2964 (18)	0.032 (5)*	
HG3_2	1.1808 (19)	0.6660 (6)	0.2125 (16)	0.028 (4)*	
C_2	1.01118 (13)	0.59836 (4)	0.42891 (11)	0.0161 (3)	
O_2	0.90772 (9)	0.62163 (3)	0.44834 (9)	0.0226 (2)	
OH_2	1.03729 (10)	0.55748 (3)	0.48645 (9)	0.0193 (2)	
HH_2	0.976 (2)	0.5538 (7)	0.550 (2)	0.045 (6)*	

### 2-(2-{[(Benzyloxy)carbonyl]amino}acetamido)-2-methylpropanoic acid (II) Atomic displacement parameters (Å^2^)

**Table:**

	*U*^11^	*U*^22^	*U*^33^	*U*^12^	*U*^13^	*U*^23^
C1	0.0193 (6)	0.0213 (6)	0.0207 (6)	−0.0080 (5)	0.0065 (5)	0.0012 (5)
C2	0.0197 (6)	0.0257 (7)	0.0206 (6)	−0.0036 (5)	0.0020 (5)	0.0004 (5)
C3	0.0259 (7)	0.0256 (7)	0.0257 (7)	−0.0064 (6)	0.0056 (6)	−0.0044 (6)
C4	0.0263 (7)	0.0212 (7)	0.0385 (8)	−0.0012 (6)	0.0107 (6)	0.0023 (6)
C5	0.0215 (7)	0.0322 (8)	0.0368 (8)	−0.0019 (6)	−0.0009 (6)	0.0102 (6)
C6	0.0227 (7)	0.0310 (7)	0.0241 (7)	−0.0107 (6)	−0.0011 (5)	0.0025 (6)
C7	0.0266 (8)	0.0227 (7)	0.0294 (7)	−0.0089 (6)	0.0121 (6)	−0.0028 (6)
O1	0.0218 (5)	0.0211 (5)	0.0237 (5)	−0.0070 (4)	0.0022 (4)	−0.0006 (4)
C	0.0127 (6)	0.0159 (6)	0.0204 (6)	0.0032 (4)	0.0028 (5)	−0.0015 (4)
O	0.0193 (5)	0.0266 (5)	0.0186 (5)	−0.0008 (4)	0.0012 (3)	0.0018 (4)
N_1	0.0144 (5)	0.0170 (5)	0.0160 (5)	−0.0009 (4)	0.0012 (4)	−0.0014 (4)
CA_1	0.0101 (6)	0.0188 (6)	0.0193 (6)	−0.0005 (5)	0.0024 (5)	−0.0010 (5)
C_1	0.0115 (6)	0.0149 (6)	0.0150 (5)	−0.0001 (4)	0.0016 (4)	0.0024 (4)
O_1	0.0089 (4)	0.0206 (4)	0.0194 (4)	0.0003 (3)	0.0008 (3)	−0.0027 (3)
N_2	0.0072 (5)	0.0183 (5)	0.0186 (5)	0.0014 (4)	0.0002 (4)	−0.0017 (4)
CA_2	0.0119 (5)	0.0164 (6)	0.0177 (6)	0.0014 (4)	−0.0006 (4)	−0.0020 (5)
CB_2	0.0150 (6)	0.0227 (6)	0.0249 (7)	−0.0031 (5)	−0.0022 (5)	−0.0048 (5)
CG_2	0.0223 (7)	0.0194 (6)	0.0242 (6)	0.0022 (5)	0.0002 (5)	0.0025 (5)
C_2	0.0137 (6)	0.0198 (6)	0.0148 (6)	−0.0004 (5)	−0.0025 (4)	−0.0031 (4)
O_2	0.0141 (4)	0.0288 (5)	0.0250 (5)	0.0056 (4)	0.0014 (3)	−0.0031 (4)
OH_2	0.0170 (4)	0.0220 (5)	0.0190 (4)	0.0000 (3)	0.0025 (4)	0.0025 (4)

### 2-(2-{[(Benzyloxy)carbonyl]amino}acetamido)-2-methylpropanoic acid (II) Geometric parameters (Å, º)

**Table:**

C1—C6	1.388 (2)	CA_1—C_1	1.5296 (16)
C1—C2	1.3929 (19)	CA_1—H1_1	0.966 (16)
C1—C7	1.502 (2)	CA_1—H2_1	0.987 (16)
C2—C3	1.386 (2)	C_1—O_1	1.2351 (15)
C2—H2	0.972 (18)	C_1—N_2	1.3322 (16)
C3—C4	1.386 (2)	N_2—CA_2	1.4662 (15)
C3—H3	0.996 (18)	N_2—H_2	0.85 (2)
C4—C5	1.384 (2)	CA_2—CG_2	1.5203 (17)
C4—H4	0.95 (2)	CA_2—CB_2	1.5314 (17)
C5—C6	1.390 (2)	CA_2—C_2	1.5360 (17)
C5—H5	0.919 (18)	CB_2—HB1_2	0.973 (17)
C6—H6	0.951 (17)	CB_2—HB2_2	0.987 (19)
C7—O1	1.4583 (16)	CB_2—HB3_2	0.966 (18)
C7—H71	0.963 (18)	CG_2—HG1_2	0.975 (18)
C7—H72	0.978 (18)	CG_2—HG2_2	0.948 (19)
O1—C	1.3431 (16)	CG_2—HG3_2	0.984 (18)
C—O	1.2274 (16)	C_2—O_2	1.2061 (15)
C—N_1	1.3403 (16)	C_2—OH_2	1.3336 (16)
N_1—CA_1	1.4449 (16)	OH_2—HH_2	0.89 (2)
N_1—H_1	0.92 (2)		
			
C6—C1—C2	119.27 (13)	C_1—CA_1—H1_1	110.9 (9)
C6—C1—C7	121.31 (13)	N_1—CA_1—H2_1	108.3 (9)
C2—C1—C7	119.38 (13)	C_1—CA_1—H2_1	107.7 (9)
C3—C2—C1	120.44 (13)	H1_1—CA_1—H2_1	106.9 (13)
C3—C2—H2	120.6 (10)	O_1—C_1—N_2	123.49 (11)
C1—C2—H2	118.9 (10)	O_1—C_1—CA_1	120.93 (11)
C2—C3—C4	119.99 (14)	N_2—C_1—CA_1	115.56 (10)
C2—C3—H3	120.7 (10)	C_1—N_2—CA_2	122.06 (10)
C4—C3—H3	119.3 (10)	C_1—N_2—H_2	119.0 (11)
C5—C4—C3	119.85 (14)	CA_2—N_2—H_2	118.6 (11)
C5—C4—H4	120.3 (12)	N_2—CA_2—CG_2	110.09 (10)
C3—C4—H4	119.8 (12)	N_2—CA_2—CB_2	109.22 (10)
C4—C5—C6	120.26 (14)	CG_2—CA_2—CB_2	109.77 (11)
C4—C5—H5	121.0 (11)	N_2—CA_2—C_2	110.42 (10)
C6—C5—H5	118.7 (11)	CG_2—CA_2—C_2	111.90 (10)
C1—C6—C5	120.16 (14)	CB_2—CA_2—C_2	105.30 (10)
C1—C6—H6	119.9 (10)	CA_2—CB_2—HB1_2	111.0 (10)
C5—C6—H6	119.9 (10)	CA_2—CB_2—HB2_2	109.4 (11)
O1—C7—C1	109.05 (11)	HB1_2—CB_2—HB2_2	108.2 (14)
O1—C7—H71	103.7 (10)	CA_2—CB_2—HB3_2	110.5 (10)
C1—C7—H71	111.4 (10)	HB1_2—CB_2—HB3_2	109.8 (14)
O1—C7—H72	108.1 (10)	HB2_2—CB_2—HB3_2	108.0 (15)
C1—C7—H72	112.4 (11)	CA_2—CG_2—HG1_2	111.5 (10)
H71—C7—H72	111.7 (14)	CA_2—CG_2—HG2_2	111.3 (11)
C—O1—C7	118.40 (11)	HG1_2—CG_2—HG2_2	107.5 (15)
O—C—N_1	123.65 (12)	CA_2—CG_2—HG3_2	108.1 (10)
O—C—O1	125.54 (12)	HG1_2—CG_2—HG3_2	110.8 (14)
N_1—C—O1	110.80 (11)	HG2_2—CG_2—HG3_2	107.6 (15)
C—N_1—CA_1	119.15 (10)	O_2—C_2—OH_2	124.28 (12)
C—N_1—H_1	118.8 (11)	O_2—C_2—CA_2	123.55 (11)
CA_1—N_1—H_1	120.7 (11)	OH_2—C_2—CA_2	111.81 (10)
N_1—CA_1—C_1	111.11 (10)	C_2—OH_2—HH_2	107.8 (13)
N_1—CA_1—H1_1	111.7 (9)		

### 2-(2-{[(Benzyloxy)carbonyl]amino}acetamido)-2-methylpropanoic acid (II) Hydrogen-bond geometry (Å, º)

**Table:**

*D*—H···*A*	*D*—H	H···*A*	*D*···*A*	*D*—H···*A*
N_1—H_1···O_1^i^	0.92 (2)	2.12 (2)	3.0298 (14)	168.7 (16)
N_2—H_2···O_1^ii^	0.85 (2)	2.09 (2)	2.9304 (15)	167.3 (18)
O*H*_2—H*H*_2···O^iii^	0.88 (2)	1.82 (2)	2.6789 (14)	162 (2)

Symmetry codes: (i) −*x*+2, −*y*+1, −*z*; (ii) *x*+1/2, *y*, −*z*+1/2; (iii) −*x*+2, −*y*+1, −*z*+1.
