# Supplementary material for: Crystal structures of Z–Gly–Aib–O−·0.5Ca2+·H2O and Z–Gly–Aib–OH
Source: Acta Crystallogr E Crystallogr Commun. 2018 Jul 27;74(Pt 8):1173–7. doi: 10.1107/S2056989018010745 (PMC6073005; doi:10.1107/S2056989018010745)
Supplement: Supplementary file 4 [file e-74-01173-sup4.pdf]

Supporting information

The crystal structures of Z-Gly-Aib-O^{-} x ½Ca^{2+} x H_2O and Z-Gly-Aib-OH

Renate Gessmann,[a] Hans Brückner[b] and Kyriacos Petratos[a]
[a]IMBB-FoRTH, 70013 Heraklion, Greece, and [b]Department of Food Sciences, Interdisciplinary Research Center, Justus-Liebig-University of Giessen, 35392 Giessen, Germany

Correspondance email: petratos@imbb.forth.gr

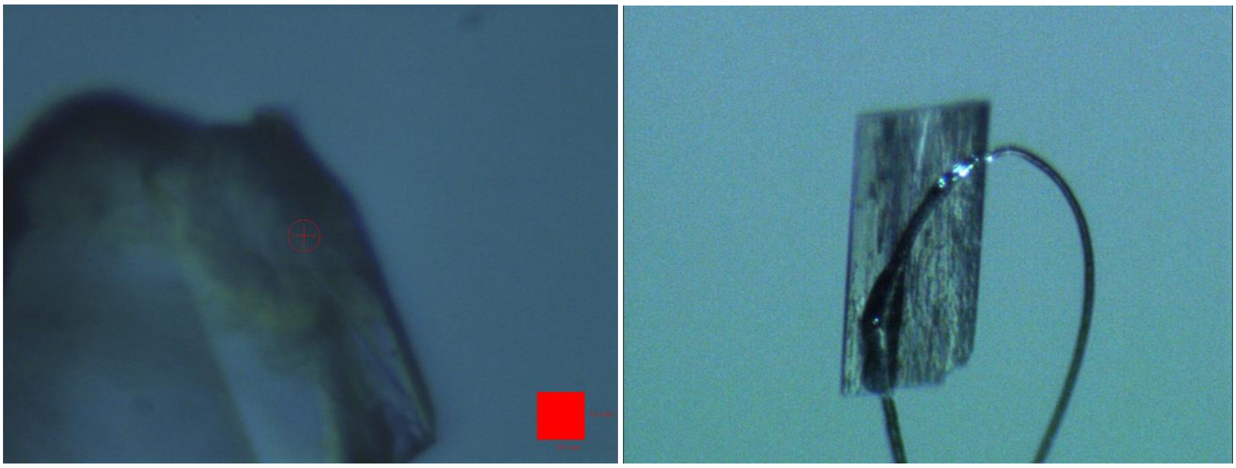

Figure **S1**.The crystals used for data collection.
Left: The complex of the dipeptide with Ca^{2+} and H_2O (Z-Gly-Aib-O^{-} x ½Ca^{2+} x H_2O, **I**).
Right: The free form of the dipeptide (Z-Gly-Aib-OH, **II**).

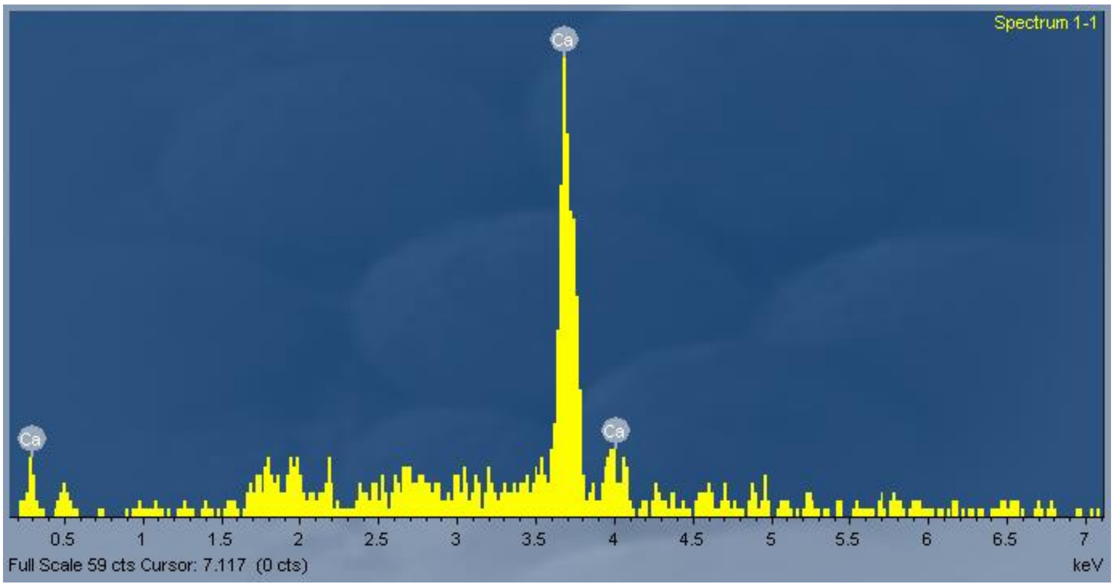

Figure **S2**. Energy-dispersive X-ray emission spectrum of a crystal selected from the same crystallization batch as the one that provided structure **I** showing peaks at 0.3, 3.7 and 4.0 keV, which are identified as the L, Kα and Kβ lines of calcium.
